# Continuous Femoral Nerve Block versus Intravenous Patient Controlled Analgesia for Knee Mobility and Long-Term Pain in Patients Receiving Total Knee Replacement: A Randomized Controlled Trial

**DOI:** 10.1155/2014/569107

**Published:** 2014-08-28

**Authors:** Lihua Peng, Li Ren, Peipei Qin, Jing Chen, Ping Feng, Haidan Lin, Min Su

**Affiliations:** ^1^The Department of Anesthesia and Pain Medicine, The First Affiliated Hospital of Chongqing Medical University, No. 1 Road Youyi Road, Yuanjiagang Community, Yuzhong District, Chongqing 400016, China; ^2^Institute of Clinical Trials, The First Affiliated Hospital of Chongqing Medical University, China; ^3^The Department of Rehabilitation Medicine, The First Affiliated Hospital of Chongqing Medical University, China

## Abstract

*Objectives*. To evaluate the comparative analgesia effectiveness and safety of postoperative continuous femoral nerve block (CFNB) with patient controlled intravenous analgesia (PCIA) and their impact on knee function and chronic postoperative pain. *Methods*. Participants were randomly allocated to receive postoperative continuous femoral nerve block (group CFNB) or intravenous patient controlled analgesia (group PCIA). Western Ontario and McMaster Universities Osteoarthritis Index (WOMAC) scores for knee and incidence of chronic postoperative pain at 3, 6, and 12 months postoperatively were compared. postoperative pain and salvage medication at rest or during mobilization 24 hours, 48 hours, and 7 days postoperatively were also recorded. *Results*. After discharge from the hospital and rehabilitation of joint function, patients in group CFNB reported significantly improved knee flexion and less incidence of chronic postoperative pain at 3 months and 6 months postoperatively (*P* < 0.05). Analgesic rescue medications were significantly reduced in patients receiving CFNB (*P* < 0.001 and *P* = 0.031, resp.). *Conclusion*. With standardized rehabilitation therapy, continuous femoral nerve block analgesia reduced the incidence of chronic postoperative pain, improved motility of replaced joints, and reduced the dosages of rescue analgesic medications, suggesting a recovery-enhancing effect of peripheral nerve block analgesia.

## 1. Introduction

Major knee surgeries have been practiced widely to cure joint dysfunction, deformity, and pain [1]. However, postoperative recovery is often accompanied by moderate-to-severe pain which hinders joint function rehabilitation. Traditionally, the methods for analgesia after major knee surgeries included oral analgesics, intramuscular injection, and intravenous administration of nonsteroidal anti-inflammatory drug (NSAIDs) or systemic opioids. These techniques were associated with the risk of nausea and vomiting, urinary retention, hypotension, and respiratory depression [2].

To maximize the analgesic efficacy and minimize its side effects, a multimodal analgesic regimen was recommended for patients who underwent knee surgery. Among a variety of analgesic techniques, peripheral nerve block analgesia has emerged as a cornerstone within this regimen, which has been recommended by the American Association of Hip and Knee Surgeons for postoperative pain management [3].

Knee is innervated primarily by femoral nerve, then by sciatic nerve, and very partially by obturator nerve (from anterior divisions of lumbar plexus); therefore, peripheral nerve block often targets femoral nerve. Femoral nerve block (FNB) is the most common type of peripheral nerve block for analgesia after major knee surgeries [4]. A set of studies have identified the superior analgesia of FNB over systematic medications in reducing morphine consumption and pain intensity. It also attained comparable analgesic efficacy as epidural analgesia with less adverse events such as hypotension and urinary retention [5].

Apart from pain, joint function is also a major concern after major knee surgery. All of these patients should undergo a process of rehabilitation, during which course, a considerable portion of patients still complained of articular pain at rest or in motion [6–8]. Few studies have been conducted to evaluate the long-term impact of peripheral nerve block on chronic postoperative pain (CPOP) and joint function especially in Asian ethnic population. We therefore conducted a randomized controlled trial to address this question.

## 2. Material and Methods

This study was approved by the Institutional Review Board of the First Affiliated Hospital of Chongqing Medical University. The protocol design was in accordance with Consolidated Standards of Reporting Trials (CONSORT) statement [9]. All potentially eligible participants were asked to give written informed consent before they were enrolled in this study. Inclusion criteria included men and women over 18 years old and younger than 75 years old who had received selective unilateral knee replacement. Exclusion criteria included bilateral knee replacement, the secondary knee revision and knee surgery not interfering with articular joint cavity (wound debridement and suture), American Society of Anesthesiology (ASA) classification of anesthesia risk IV and V grades, body mass index higher than 35, coagulation dysfunction, activated partial thromboplastin time (APTT) higher than the upper limit by 10 s, prothrombin time (PT) higher than the upper limit by 5 s, International Normalized Ratio (INR) higher than 1.3 or any of the criteria met above, local infection of puncture sites, neurological diseases, uncontrolled general infection, and intraoperative cardiac arrest.

### 2.1. Sample Calculation

Based on the treatment efficacy in a pilot study, a 10% difference between the treatment (CFNB) and control (PCIA) groups in the incidence of moderate-to-severe pain in motion 3 months after the surgery was used to estimate the sample size required in this study. Statistical power of 80% at the 0.05 significance level required 140 subjects in each group, assuming a 15% dropout rate. A total of 280 patients were randomly allocated in a 1 : 1 ratio to either the CFNB group or the PCIA group. Statistics Analysis System software (SAS) proc plan procedure was used to generate the random number. A sealed opaque envelope, which contained the group allocation, was prepared for each patient. The envelope was not opened until the patient was enrolled in the study.

All participants were informed with the two methods of patient controlled analgesia and the tool of evaluating pain (visual analogue scale for pain); antibiotics were used for prophylaxis preoperatively, intraoperatively, and postoperatively. The protocol of prophylaxis for thromboembolism in patients receiving continuous femoral nerve block was initiated 12 hours postoperatively with subcutaneous injection of low molecular weight heparin (LMWH), 8 hours before the femoral catheter was estimated to be extracted; the use of LMWH was paused. Patients receiving intravenous patient controlled analgesia received LMWH from 12 hours postoperatively till the hospital discharged all participants.

### 2.2. Procedure of Anesthesia and Postoperative Analgesia Procedures

#### 2.2.1. Types of Anesthesia and Surgery

All of the participants received general anesthesia with intravenous and inhalational anesthetics. They were monitored with electrocardiogram (ECG), heart rate (HR), SpO_2_, and invasive arterial blood pressure monitoring; HR was maintained at 60–100 beats/minute, SpO_2_ at 98–100%, and mean arterial pressure (MAP) at 65–80 mmHg. Fluid therapy was administered at 5–10 mL/kg/h; vasodilators, vasoconstrictors, and diuretics were used when necessary. The anesthetics used were midazolam 0.1~0.15 mg/kg (but etomidate 0.15~0.2 mg/kg for the patients over 65 years old), propofol 2.0~2.5 mg/kg, sufentanil citrate 0.3~1.0 *μ*g/kg, and vecuronium 0.08~0.12 mg/kg for anesthesia induction. The maintenance of anesthesia was with inhalation of 1%~3% sevoflurane and continuous intravenous infusion of remifentanil 7~8 *μ*g/kg/h and propofol 25~75 *μ*g/kg/min with microperfusion pump. Anesthetic depth was monitored with Narcotrend index (NI, MT Monitor Technik, Bad Bramstedt, Germany) to maintain a Narcotrend index between 40 and 50. In order to reduce the bleeding during the total knee arthroplasty (TKA), pneumatic tourniquet was used for operation with the pressure of 45 Kpa and pneumatic tourniquet time was recorded. All surgical procedures included selecting medial patella incision, using the medial-quadriceps-tendon-patellar approaches, fixing artificial knee joint with bone cement, washing the articular wound with hydrogen peroxide, iodine solution, and saline, taking out soft tissue and bone debris, and assembling drainage before closing. Upon closure of the wound, 5–10 *μ*g sufentanil was intravenously injected, and loading dose of PCA was injected. Participants were assessed as fulfilling the discharge criteria of being discharged from postanesthetic care unit (PACU), if Steward score was larger than 4 and VAS for pain was less than 4 [10]. The surgeons wrapped the limb with elastic bandage in order to prevent deep venous thrombosis. The drainage was removed on 24 hours postoperatively, while mobilization was initiated within 4 h after the surgery. Two surgical teams with four surgeons in total and two anesthesiologists irrelevant with the assessment of the outcomes were responsible for the surgery and anesthesia; practicing years and surgical time were comparable between these two groups.

#### 2.2.2. Types of Postoperative Analgesia

The femoral nerve block was performed before the induction of anesthesia on the leg to be operated on. All procedures were performed by one group of senior anesthesiologists. All of the patients were placed in supine position with their legs extended. After sterile preparation, puncture site was identified using ultrasound guidance (2 cm distal to the inguinal ligament and 1~2 cm lateral to the femoral artery). 2% lidocaine was used for topical anesthesia; then an insulated needle (Contiplex B Braun, Melsungen, Germany) (20 G∗45 mm, short bevel, 30°) was connected to the nerve stimulator (Innervator, Fisher & Paykel, New Zealand). The parameters were as follows: stimulating intensity of 1 mA at a rate of 2 Hz. The needle was advanced at 30°~45° angle to the skin, until quadriceps femoral muscle twitches were elicited. Its position was accepted if contractions were still elicited when an output was equal to 0.3 mA. Then, the patients were given 10 mL 2% lidocaine and 10 mL 1% ropivacaine as an initial dose. The catheter remained in place for 10~15 cm. When catheter insertion was difficult, 0.9% saline solution was injected through the cannula to ease the catheter placement. To confirm the correct position of the catheter, the cutaneous sensation in the area of the femoral nerve was assessed using a cold test [11]. 30 minutes before the end of the operation, the catheter was connected to the PCA pump; the patients received a loading dose of 5 mL of 0.15% ropivacaine followed by an infusion of 0.15% ropivacaine at 5 mL/h, with bolus of 5 mL and lock time of 30 min. The catheter insertion site was checked once daily for signs of infection or blood through the catheter. Preoperatively, a loading dose of 30 mL was injected for intraoperative analgesia. For participants in PCIA group, the formula of the PCIA included tramadol 800 mg, flurbiprofen axetil 100 mg, and dexamethasone 5 mg with saline added up to a volume of 80 mL in total; they received a loading dose of 2 mL followed by an infusion rate of 1 mL/h with bolus of 2 mL; the lock time was set at 15 min. All participants received the preventive use of antiemetic as intravenous injection of 4 mg ondansetron.

#### 2.2.3. Analgesia Rescue Protocol

**Figure 1:**
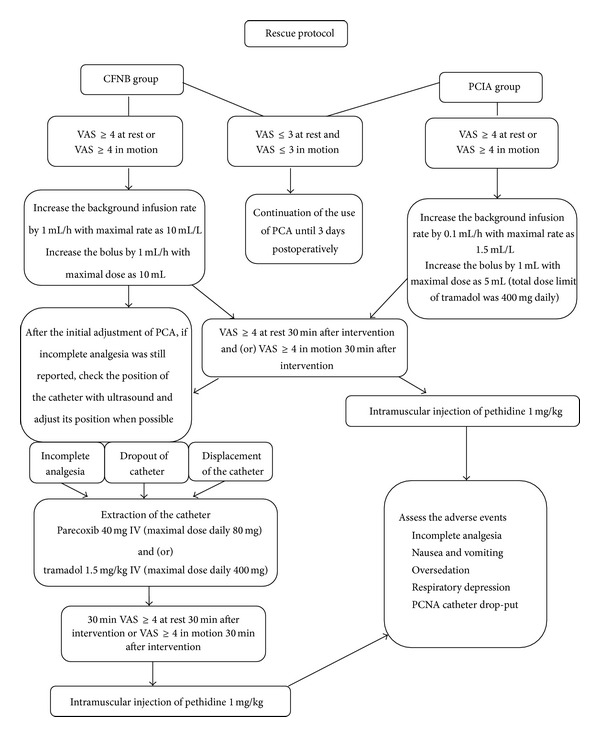
The regimen of analgesic rescue for patients in both groups. A “three-ladder” analgesic rescue protocol was designed: first, the adjustment of PCA parameters and checking of the femoral catheter in CFNB group; second, use of nonopioid analgesic medications; third, use of opioid analgesic medications. PCA was the abbreviation for patient controlled analgesia, CFNB for continuous femoral nerve block, PCIA for patient controlled intravenous analgesia, and VAS for visual analogue scale.

A strict rescue regimen of analgesia was adopted. During the implantation of patient controlled analgesia, a three-stage rescue “ladder” was adopted: the anesthesiologists decided on, first, the alternation of the PCA pump parameters with inspection of the catheter, second, the use of intravenous nonopioid rescue analgesics, and, finally, the use of opioid rescue medication (pethidine, intramuscular, 0.5–1 mg/kg) when nonopioid rescue medications were deemed as ineffective (visual analogue scale for pain larger than 3) 30 minutes after the intravenous injection. For patients receiving intravenous patient controlled analgesia, the alternation of the parameters of PCA pumps was considered first and then a salvage intravenous injection of tramadol within the daily dose limitation (400 mg daily); then pethidine (intramuscular, 0.5–1 mg/kg) was used when nonopioid rescue medications were deemed as ineffective (visual analogue scale for pain larger than 3) 30 minutes after the intravenous injection; see Figure 1.

#### 2.2.4. Preventive Measures of Analgesia-Related Adverse Events

All participants included in this study received the preventive use of antiemetic as an intravenous injection of 4 mg ondansetron. During the hospital stay, one team specializing in providing acute pain service (APS) to the participants inspected the ward every 4 hours to assess the adverse events.

*Incomplete Analgesia*. See rescue regimen of analgesia.

*Nausea and Vomiting*. Intramuscular injection of 10 mg metoclopramide or intravenous injection of 4 mg ondansetron, 0.1 mg/kg of ondansetron was the maximal dose.

*Oversedation*. Reduce the background infusion by 0.1–0.5 mL/h in PCIA group and 0.5–2 mL/h in CFNB group, with an intravenous injection of 0.4–0.8 mg naloxone.

*Respiratory Depression*. Reduce the background infusion by 0.1–0.5 mL/h in PCIA group and 0.5–2 mL/h in CFNB group, with an intravenous injection of 0.4–0.8 mg naloxone; extract the catheters of PCIA and CFNB pump if necessary, with mask ventilation with 3–10 L/min, mechanical ventilation with endotracheal intubation if necessary, and cardiopulmonary resuscitation if necessary.

*Catheter Loss of CFNB*. Extract the catheter and switch to PCIA.

*Local Hematoma of CFNB Catheter*. Compress with a sand bag and switch to PCIA.

*Local Infection of CFNB Catheter*. Extract the catheter and switch to PCIA.

*Topic Pain of CFNB Catheter*. Fix the catheter with suitable suture, with the topical use of diclofenac sodium ointment.

*Fall*. A Bromage scale was used to assess the muscle strength of the lower limb. It is a four-point scale, with 1 meaning no motor block, 2 meaning unable to raise the legs, 3 meaning unable to bend the knee, and 4 meaning unable to bend the knee. Three/four grade is an indication of motor block and it is advised to switch from CFNB to PCIA. Escort and surveillance of the patients are mandatory when knee joint exercise was carried out.

*Prophylaxis of Thromboembolic Events*. The protocol of prophylaxis for thromboembolism in patients receiving continuous femoral nerve block was initiated 12 hours postoperatively with subcutaneous injection of low molecular weight heparin (LMWH). Eight hours before the femoral catheter was estimated to be extracted, the use of LMWH was paused. Patients receiving intravenous patient controlled analgesia received LMWH from 12 hours postoperatively; the injection of LMWH was practiced till the hospital discharged all participants. On day 3 and day 7 postoperatively, all patients were monitored with lower vascular ultrasound to assess the thromboembolic events.

### 2.3. Rehabilitation Program

On day of surgery, all participants were advised to start a deep breathing exercise and an active ankle range of motion (ROM) exercise at the tolerance of the patients, on postoperative day 1, lower-limb isometric exercise (quadriceps, hamstrings, and gluteal sets), passive ROM training of Patella, and active ROM exercise of hip of at least 2 hours per day. From postoperative day 2 to day 3, continuous passive motion (CPM) of knee begins at 0 degree to 45 degrees, reaching 60 degrees in the first week, gradually reaching 90 degrees in 3 weeks; the duration of exercise was at least 2 hours per day. From postoperative day 4 to week 2, participants were advised to start CPM of knee of at least 1 hour per day and, simultaneously, active-assisted ROM of knee as tolerated of at least 2 hours per day. From postoperative week 3 to week 6, participants were advised to start isotonic and isometric muscle strengthening training of at least 4 hours per day, especially the quadriceps and straight leg raising exercise. From postoperative week 7 to week 12, weight-bearing of at least 2 hours per day was begun in this period, according the patient's situation with different ambulatory aids, for example, walker, crutches, and cane. Between postoperative week 13 and week 20, participants were advised to start resisted strength training of at least 1 hour per day, ROM exercise of at least 2 hours per day, and weight-bearing without aids of at least 2 hours per day.

### 2.4. Outcome Assessment

Chronic postoperative pain (CPOP) was diagnosed according to the definition of International Association of Study on Pain (IASP) [12]. This study added the use of numerical rating scale (NRS≧1) as the criterion for assessing CPOP. The primary outcome was incidence of moderate-to-severe pain at 3 months postoperatively; the samples of the study were calculated according to this outcome, which was defined as NRS for pain ≧4. The outcome was evaluated at rest (lie supine) or in motion (walk without aid on level ground for 5 minutes). During hospitalization, the severity of pain was assessed with visual analogue scale (at rest and in motion), pain at rest was defined when the patient was lying supine in bed, and pain in motion was rated when the most severe pain occurred during passive motion and active exercise within a day (after analgesic rescue administration). Time points for assessing pain were 24 hours, 48 hours, 7 days, 3 months, 6 months, and 12 months postoperatively. WOMAC scales for knee function were assessed during admission and 3, 6, and 12 months postoperatively.

Bolus frequency, rescue frequency, mean doses of rescue medications, and adverse events related to patient controlled analgesia were recorded. Baseline characteristics were categorized into preoperative (age, weight, height, education, gender, ASA grading, and bilateral or unilateral knee replacement) and intraoperative (surgical time, anesthesia time, tourniquet time, and doses of opioid analgesics) categories. Degrees of flexion of the knee were assessed by calibrating the angle of the extended line of the femur and the tibias by an independent orthopedic doctor.

### 2.5. Statistical Methods

All analyses were performed using the SAS 9.2 software package. The level of statistical significance was set at 0.05. Data were expressed as mean± standard deviation (SD) for continuous variables and total number (percent frequency) for categorical variables. A group *t*-test, Wilcoxon rank test, or Kruskal-Wallis test was used to compare results for continuous variables. Chi-square test was used to compare results for categorical variables. Fisher's exact test was used for categorical variables when the number of events was less than 5. Both intention-to-treat analysis (ITT analysis) and per-protocol set analysis (PPS analysis) were used in this study. Statistical difference was assumed when *P* value <0.05.

## 3. Results

**Figure 2:**
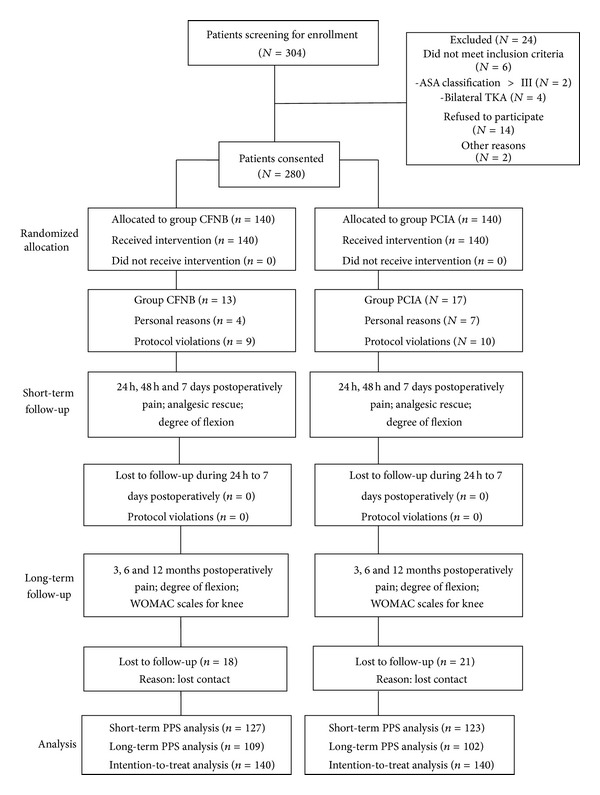
Study flow diagram following the CONSORT statement.

A total of 304 patients were screened for enrollment; 24 patients were excluded for not meeting eligible criteria or refusing to participate. The remaining 280 participants were included and randomized into a CFNB group in which continuous femoral nerve block was administered (*N* = 140) and a PCIA group in which intravenous patient controlled analgesia was used (*N* = 140); 13 participants in the CFNB group and 17 in the PCIA group were excluded from the PPS analysis for short-term outcome because of personal reasons or rescue protocol violation. Thirty-nine participants were lost to follow-up 3 months postoperatively and were not included in the PPS analysis of long-term follow-up. The principle of per-protocol analysis was followed in the analysis of preoperative characteristics. The two groups were comparable in baseline characteristics (age, weight, preoperative WOMAC scores, and ASA grading); surgical time and anesthetic were also similar in the two groups (Table 1). Hypertension, coronary artery disease, chronic obstructive pulmonary disease, and diabetes are common types of preoperative complications in both groups (Table 2). A diagram following CONSORT statement was presented below (Figure 2).

### 3.1. Incidence of Chronic Postoperative Pain (CPOP)

In the PPS analysis, the incidence of chronic postoperative pain was 68.9% in PCIA group and 47.7% in CFNB group at 3 months postoperatively (*P* = 0.002); CPOP was still observed in PCIA group at 6 months with the reported rate of 52.4%, while that of CFNB group was 33.0% (*P* = 0.004). At 12 months postoperatively, the incidence of CPOP in both groups was minimal and no statistical difference was found (*P* = 0.224). ITT analysis still found a significantly reduced incidence of CPOP in CFNB group compared with PCIA group (Table 3).

The incidence of chronic postoperative moderate-to-severe pain was evaluated at 3 months, 6 months, and 12 months. In PPS analysis, the incidence of moderate-to-severe pain in motion was 34.8% in CFNB group and 46.0% in PCIA group at 3 months postoperatively (*P* < 0.05). At 6 months postoperatively, this rate in group CFNB dropped to 22.9% while that of group PCIA decreased to 36.3% (*P* < 0.05). At 12 months postoperatively, this rate decreased to 1.4% in CFNB group and to 4.3% in PCIA group (*P* = 0.214). In ITT analysis, no statistical difference was found between the two groups (Table 4).

### 3.2. Pain Scores and Analgesic Rescue Medications

In PPS analysis, no statistical difference was found in preoperative VAS scores after the administration of analgesic rescue medications between these two groups 24 hours and 48 hours postoperatively. On the 7th day postoperatively, patients in CFNB group reported significantly reduced degree of pain scores in motion (*P* < 0.0001) or at rest (*P* = 0.031). Chronic postoperative pain was assessed at 3 months, 6 months, and 12 months postoperatively; patients in CFNB group reported a significantly lower level of pain intensity at 3 months in motion (*P* = 0.025) or at rest (*P* < 0.0001) and at 6 months (*P* = 0.011 for pain in motion and *P* < 0.0001 for pain at rest) postoperatively but not at 12 months postoperatively (Table 5).

### 3.3. Dose of Analgesic Medications and Analgesic Rescue

During the PCA phase, patients in CFNB group required less frequency of analgesic bolus and rescue (*P* = 0.003 and 0.002, resp.). During the PCA period, two types of rescue analgesic medications (parecoxib and tramadol) were consumed less in CFNB group (*P* = 0.022 and 0.000, resp.). In the non-PCA period, all analgesic medications (parecoxib; paracetamol; tramadol; flurbiprofen) were consumed less in CFNB group (Table 6).

### 3.4. WOMAC Knee Scores and Knee Flexion

**Figure 3:**
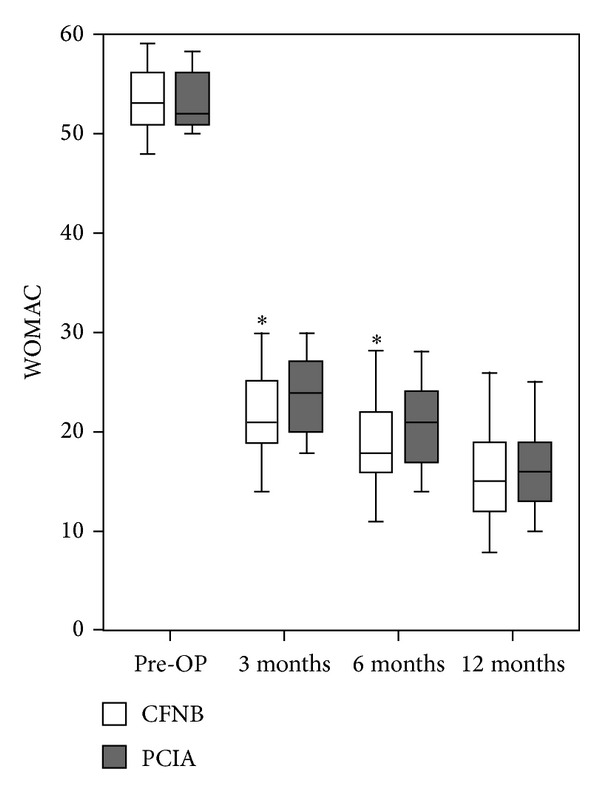
WOMAC scores for knee before and after surgery. *A significant difference between group CFNB and group PCIA, *P* < 0.05. WOMAC was the abbreviation for Western Ontario and McMaster Universities Osteoarthritis Index, CFNB for continuous femoral nerve block, and PCIA for patient controlled intravenous analgesia.

**Figure 4:**
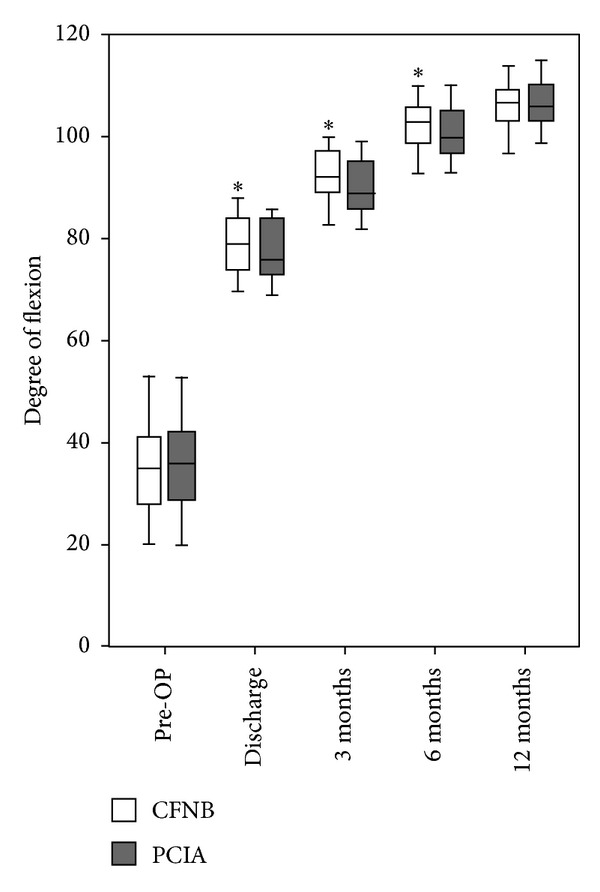
Degree of flexion for knee before and after surgery. *A significant difference between group CFNB and group PCIA, *P* < 0.05. CFNB was the abbreviation for continuous femoral nerve block and PCIA for patient controlled intravenous analgesia.

The day before surgery, WOMAC knee scores and degree of knee flexion were comparable between the two groups. Participants receiving continuous femoral nerve block reported slightly improved knee function at 3 months and 6 months postoperatively (*P* = 0.014 and 0.011, resp.) (Figure 3); the same trend was observed for flexion of knee (Figure 4).

### 3.5. PCA-Related Adverse Events

Before the use of analgesic rescue, incomplete analgesia was the major adverse event reported, with 7.08% in CFNB group and 17.07% in PCIA group (*P* = 0.015). The incidence of other adverse events varied from 0 to 4.06% (oversedation; nausea and vomiting; respiratory depression; muscle weakness). Nerve-block-catheter-related adverse events, such as catheter thrombosis, catheter dropout, local hematoma, and local infection, were not observed in CFNB group. During the non-PCA period, the incidence of incomplete analgesia was slightly higher in PCIA group (20.3% versus 11.8%, *P* = 0.066) (Table 7).

## 4. Discussion

The prominent finding of this study was that continuous femoral nerve block postoperatively could effectively reduce the incidence of postoperative chronic pain and improve joint function during short-term and long-term follow-up. The use of CFNB was also associated with less analgesic medications for rescue, indicating the advantage of peripheral nerve block over intravenous patient controlled analgesia in controlling acute perioperative pain and the development of chronic postoperative pain. In this study, we observed less severity of pain and slightly improved degree of flexion on 7 days postoperatively, suggesting a rehabilitation-enhancing effect than continuously intravenous infusion of analgesics, which reinforced the evidence from other randomized controlled trials that femoral nerve block could bring good quality of postoperative analgesia compared to systemic analgesics or epidural analgesia and thus fasten the recovery process and allow patients to be more mobile after total knee arthroplasty [13, 14].

### 4.1. Rationality of Analgesic Medication

Different volumes and concentrations of local anesthetics could impact the analgesic efficacy and motor block of peripheral continuous nerve block analgesia; most of the previous studies adopted the concentrations from 0.2% to 0.3% of ropivacaine [15–17]. To avoid motor block and reduce the risk of fall [18, 19], 0.15% of ropivacaine was used for femoral catheter infusion in this study. No cases of falls were recorded. Bromage scores were good except for 5 participants (4 in CFNB group and 1 in PCIA group). For participants in PCIA group, nonopioid medications (tramadol and NSAID) were used; several large-sampled meta-analyses have demonstrated that tramadol could attain satisfactory analgesic effects with well-organized acute pain service [20]. The major adverse events associated with nonopioid medications were nausea and vomiting [21]; therefore, a combined prevention regimen with ondansetron and dexamethasone was used. This study also demonstrated that NSAID could be safely used for controlling inflammatory pain after knee surgery while avoiding opioid-related adverse events, such as respiratory depression, pruritus, and oversedation [22, 23].

One feature of this study was that a strict analgesic rescue protocol was designed to minimize the number of patients with incomplete analgesia, with the transition analgesia from PCA period to non-PCA period. During the non-PCA period, participants in CFNB group required less analgesic medications after cessation of patient controlled analgesia; less incidence of incomplete analgesia was also observed in CFNB group, suggesting a more efficacious role of peripheral nerve block in ameliorating the transition of acute surgical pain to persistent pain during hospital recovery [24, 25].

### 4.2. Chronic Postoperative Pain and Function

As the primary outcome for this study, less incidence of chronic postoperative pain was found at 3 and 6 months postoperatively but not at 12 months; the same trend was observed for WOMAC scores for knee [26–28]. WOMAC scores for knee and flexion were two important parameters for the evaluation of knee function; three dimensions were assessed with WOMAC scores: pain, joint stiffness, and daily life ability [29]. Besides modes of postoperative exercise [30, 31], postoperative analgesia could also impact the knee function [32]. With a unified exercise protocol, we still observed a better improvement of joint function in participants receiving CFNB; this finding confirms the beneficial role of peripheral nerve block analgesia in knee surgery.

### 4.3. Limitations

Several limits existed in this study. First, we found a statistically significant yet not clinically important increase in the degree of flexion 7 days postoperatively, which weakened the power of this study; second, we observed the chronic pain and WOMAC by subjective interviews without direct calibration of joint movement; NRS for pain was recorded regardless of the use of analgesics; third, blindness was not possible for the participants and anesthesiologists for the CFNB group.

### 4.4. Conclusion

In conclusion, after rigorous analgesic rescue protocol, CFNB revealed advantage over intravenous patient controlled analgesia in reducing the dosage of rescue medications and improving short-term and long-term joint function, reducing incidence of chronic postoperative pain.

## Figures and Tables

**Table 1:** Baseline Characteristics of eligible patients^#^.

Parameters	CFNB (*N* = 140)	PCIA (*N* = 140)	*P* value
Age	66.81 ± 9.41	68.03 ± 11.17	0.323
Height (cm)	158.20 ± 7.69	159.41 ± 9.39	0.240
Weight (kg)	62.99 ± 8.38	63.17 ± 7.35	0.847
WOMAC scores for knee	53 (51–56)	54 (51–56)	0.212^△^
Surgical time (min)	87.21 ± 34.23	82.37 ± 30.95	0.216
Anesthesia time (min)	125.69 ± 32.62	121.08 ± 32.30	0.235
Blood loss (mL)	89.72 ± 93.49	88.89 ± 86.27	0.939
Tourniquet time (min)	60.41 ± 29.24	56.80 ± 26.92	0.284
Gender			
Male/female	38/102	49/91	0.156^△^
American Society of Anesthesiologists (ASA) Grading^▲^			
1	19	25	
2	59	49	0.990∗
3	62	66	

^#^Continuous variables were described as mean ± SD, categorical variables as number of events (*n*); *Kruskal-Wallis test was used; ^△^Chi square test was used; ^▲^Wilcoxon rank test was used.

The rest of parameters were compared using independent *t*-test; statistical significance was considered when *P* value <0.05.

**Table 2:** Preoperative comorbidity of enrolled participants.

Preoperative comorbidity^△^	CFNB group	PCIA group	*P* value
	*n*/*N* (%)	*n*/*N* (%)	
Cardiovascular system			
Hypertension	41/140 (34.2)	29/140 (24.2)	0.098
Coronary artery disease	7/140 (5.8)	7/140 (5.8)	1.000
Arrhythmia			
Atrial fibrillation	1/140 (0.8)	1/140 (0.8)	1.000∗
Ventricular premature	0/140 (0)	3/140 (2.5)	0.247∗
Sinus bradycardia	0/140 (0)	2/140 (1.7)	0.498∗
Respiratory system			
Asthma	2/140 (1.7)	0/140 (0)	0.498∗
COPD	4/140 (3.3)	8/140 (5.7)	0.238
Respiratory failure	1/140 (0.8)	1/140 (0.8)	1.000∗
Urological system			
Renal syndrome	0/140 (0)	1/140 (0.8)	0.500∗
Renal failure	0/140 (0)	1/140 (0.8)	0.500∗
Endocrine system and autoimmune disease			
Diabetes	18/140 (15)	9/140 (7.5)	0.068
Rheumatoid arthritis	0/140 (0)	3/140 (2.1)	0.247∗
Thromboembolic events			
Cerebral embolism	2/140 (1.7)	0/140 (0)	0.498∗
Myocardial infraction	0/140 (0)	0/140 (0)	
Venous thrombosis	0/140 (0)	2/140 (1.7)	0.498∗
Peripheral artery embolism	0/140 (0)	0/140 (0)	

^△^Categorical variables as number of events (*n*), Chi square test was used; events less than 5 were compared with Fisher's exact test, *P < 0.05.

**Table 3:** Incidence of chronic postoperative pain after TKA^#^.

Intention-to-treat analysis			
Time points	Group CFNB (*n* = 140)	Group PCIA (*n* = 140)	*P* value
3 months—*n* (%)	52 (37.1)	71 (50.7)	0.022∗
6 months—*n* (%)	36 (25.7)	54 (38.5)	0.021∗
12 months—*n* (%)	5 (3.5)	9 (6.4)	0.273

Per-protocol analysis			
Time points	Group CFNB (*n* = 109)	Group PCIA (*n* = 103)	*P*-value

3 months—*n* (%)	52 (47.7)	71 (68.9)	0.002∗
6 months—*n* (%)	36 (33.0)	54 (52.4)	0.004∗
12 months—*n* (%)	5 (4.5)	9 (8.7)	0.224

^#^Chi-square test was used for comparisons. *P < 0.05.

**Table 4:** Incidence of chronic postoperative moderate-to-severe pain in motion^#^.

Intention-to-treat analysis			
Time points	Group CFNB (*n* = 140)	Group PCIA (*n* = 140)	*P* value
3 months—*n* (%)	38 (27.1)	47 (33.5)	0.242
6 months—*n* (%)	25 (17.8)	37 (26.4)	0.084
12 months—*n* (%)	2 (1.4)	5 (4.3)	0.251

Per-protocol analysis			
Time points	Group CFNB (*n* = 109)	Group PCIA (*n* = 102)	*P* value

3 months—*n* (%)	38 (34.8)	47 (46.0)	0.047∗
6 months—*n* (%)	25 (22.9)	37 (36.3)	0.034∗
12 months—*n* (%)	2 (1.8)	5 (4.9)	0.214

^#^Chi-square test was used for comparisons.*P < 0.05.

**Table 5:** Visual analogue scale during hospitalization and postoperatively^#^.

Time point	CFNB group	PCIA group	*P* value
VAS scores in motion			
1 day preoperatively	5 (4-5)	5 (4-5)	0.494
24 h postoperatively	3 (3-4)	3.5 (3-4)	0.262
48 h postoperatively	3 (3-4)	3 (3-4)	0.143
7 days postoperatively	3 (3-4)	4 (4-4)	<0.0001∗
3 months postoperatively	3 (2–4)	3 (3-4)	0.025
6 months postoperatively	3 (2-3)	3 (3-4)	0.011
12 months postoperatively	2 (1–3)	2 (1–3)	0.581
VAS scores at rest			
1 day preoperatively	2 (1–3)	2 (1–3)	0.44
24 h postoperatively	3 (3-4)	3 (3-4)	0.211
48 h postoperatively	3 (3-4)	3 (3-4)	0.297
7 days postoperatively	3 (2-3)	3 (3-3)	0.031∗
3 months postoperatively	1 (1-2)	2 (1–3)	<0.0001
6 months postoperatively	1 (1-1)	2 (1-2)	<0.0001
12 months postoperatively	1 (1-1)	1 (1-1)	0.681

^#^Continuous variables were described as median (interquartile range) and Wilcoxon rank-sum test was used, *P value <0.05. The total number of patients was 127 in CFNB group and 123 in PCIA group for in-hospital pain evaluation, while 109 patients in CFNB group and 102 patients in PCIA group were included in the analysis of chronic pain.

**Table 6:** Doses of analgesic medications of patient controlled analgesia and rescue regimen^#^.

	Analgesic outcomes	CFNB (*N* = 127)	PCIA (*N* = 123)	*P* value
PCA period	Frequency of bolus	2.3 ± 0.8	2.6 ± 0.7	0.003∗
	Frequency of rescue	0.6 ± 0.8	1.1 ± 1.3	0.002∗
	Mean doses of rescue medication			
	Parecoxib (mg)	22.4 ± 28.4	31.5 ± 34.5	0.022∗
	Tramadol (mg)	5.1 ± 20.7	24.0 ± 42.6	0.000∗
	Pethidine (mg)	0	0.4 ± 3.2	0.158

	Mean doses of rescue medications			
	Parecoxib (mg)	963.8 ± 479.0	1141.5 ± 259.5	0.000∗
Non-PCA period	Paracetamol (mg)	166.3 ± 163.1	219.3 ± 152.9	0.009∗
	Tramadol (mg)	19.2 ± 18.8	25.3 ± 17.6	0.009∗
	Flurbiprofen (mg)	7.9 ± 18.3	14.2 ± 22.7	0.016∗

^#^Continuous variables were described as mean ± SD and independent *t*-test was used; *statistical significance was considered when *P* value <0.05.

**Table 7:** Adverse events related to patient controlled analgesia^#^.

Adverse events	CFNB (*n* = 127)	PCIA (*n* = 123)	*P* value
PCA period			
Incomplete analgesia			
In motion	9 (7.08)	21 (17.07)	0.015∗
At rest	0 (0)	0 (0)	—
Oversedation	2 (1.57)	5 (4.06)	0.275
Nausea and vomiting	3 (2.36)	4 (3.25)	0.719
Respiratory depression	0 (0)	2 (1.62)	0.149
Muscle weakness	4 (3.14)	1 (0.81)	0.370
Catheter thrombosis	0 (0)	0 (0)	—
Catheter dropout	0 (0)	0 (0)	—
Local hematoma	0 (0)	0 (0)	—
Local infection	0 (0)	0 (0)	—
Non-PCA period			
Incomplete analgesia	15 (11.8)	25 (20.3)	0.066
Oversedation	0 (0)	0 (0)	—
Nausea and vomiting	1 (0.78)	0 (0)	1.000
Respiratory depression	0 (0)	0 (0)	—

^#^For events less than 5 in both groups, Fisher's exact test was used; the rest were compared using chi-square test, *Pvalue <0.05.
